# B Cells Protect T Cell-deficient mice from Cryptococcal Brain Invasion

**DOI:** 10.1080/21505594.2017.1393601

**Published:** 2017-12-26

**Authors:** Michael J. Davis, Michail S. Lionakis

**Affiliations:** From the Laboratory of Clinical Immunology and Microbiology, National Allergy & Infectious Diseases (NIAID), National Institutes of Health (NIH), Bethesda, Maryland, USA

**Keywords:** brain, B cells, cryptococcus, IgM, T cells

In this issue of *Virulence*, Dufaud et al^1^ utilize a Rag1-deficient murine model and adoptive transfers of B-cells or IgM-containing serum to directly examine the role of B-cells in host defense against the opportunistic yeast fungus *Cryptococcus neoformans* (CN). Cryptococcal meningoencephalitis remains a leading cause of mortality in patients with HIV/AIDS resulting in ∼200,000 annual deaths worldwide,^2^ hence, better understanding of the cellular and molecular factors that protect the host from cryptococcosis could lead to immune-based therapies and better patient outcomes. The susceptibility of patients with AIDS and idiopathic CD4+ T-cell lymphocytopenia to cryptococcosis clearly underscores the critical contribution of CD4+ T-cells in effective host defense. Moreover, macrophages are critical in promoting fungal clearance following their priming and effective cross-talk with T-cells via the production of IFN-gamma.^3^ On the other hand, the role of B-cells in anti-CN host defense has remained less understood. Prior studies in C57BL/6 mice depleted of B-1 cells,^4^ in sIgM*^−/−^* mice that lack secreted IgM,^5^ and in X-linked immunodeficient mice (XID) that possess a mutation in the Bruton's tyrosine kinase (Btk),^6^ have suggested a contribution of B-1 cells or naïve serum IgM in protection against cryptococcal brain invasion; however, definitive conclusions about the direct role of B-cells in anti-CN immunity were confounded in all of the aforementioned studies by the presence of T cells and because of associated defects in cellular immunity (XID mice) and in B-cell development (sIgM*^−/−^* mice).

In the present study, the authors utilize a low-virulence strain of CN, which upon infection of C57BL/6 mice results in a significant chronic pulmonary infection with only low-level dissemination and efficient local control of the fungus in the brain.^7^ In contrast to C57BL/6 mice, Dufaud el al show that CN-infected Rag1-deficient mice (on the C57BL/6 genetic background), which lack both T and B-cells, display a marked increase in fungal proliferation in the brain, which is particularly accentuated after the second week post-infection (Figure 1B). The authors restore (at least partly) B-cell function in Rag1-deficient hosts by adoptively transferring B-cells purified from the spleens of C57BL/6 mice into the Rag1-deficient animals. This transfer restored the numbers of B-1a and B-1b cells recruited to the CN-infected lungs, but not T-cells or B-2 cells, in agreement with previous reports of B-cell adoptive transfers in other inflammatory settings.^8^ Intriguingly, this B-cell transfer led to a marked reduction in the burden of CN in the brain of these mice compared to Rag1-deficient mice that did receive B-cells, a reduction in fungal load that reached levels comparable to those seen in CN-infected C57BL/6 mice. Instead, B-cell adoptive transfers in Rag1-deficient mice did not affect the fungal load in the CN-infected lung, highlighting a brain-specific requirement for B cell-dependent control of fungal load during cryptococcosis. This brain-specific role of B-cells in protection against cryptococcosis is is on par with recent clinical reports of brain infections by CN (and other fungi including *Aspergillus*) in patients receiving the small-molecule irreversible BTK inhibitor ibrutinib, which impairs B-cell receptor signaling, although patients with inherited BTK deficiency have not been reported to develop brain cryptococcal disease thus far.^9–10^

The results presented by Dufaud and colleagues are the most definitive data to date linking B-cells and cryptococcal host defense in the brain, especially in light of the demonstrated ability of B-cells to promote protection despite the absence of T-cells. Yet, important questions remain with regard to the mechanism(s) by which the transferred B-cells exert their protective effects and reduced CN invasion in the brain. While B-cells are best known for their secretion of antibody, they also participate in immune signaling, secrete inflammatory mediators, phagocytose and kill microbes and present antigen to T-cells.^11–13^ With the exception of the latter mechanism which is not pertinent herein given that the results were obtained in Rag1-deficient mice that lack T-cells, all other mechanisms remain viable possibilities. Dufaud et al attempted to determine if the observed B cell-mediated protection was due to secreted antibody or due to other B cell-intrinsic activities. They found that adoptive transfer of and restoration of B-cells in the lungs of CN-infected Rag1-deficient mice dramatically increased the levels of IgG and IgM in Rag1-deficient mice which otherwise have undetectable levels of antibody in their serum. However, this antibody was not necessarily CN-specific as these antibodies did not recognize GXM, the major CN-capsule antigen; future work will be needed to determine whether these antibodies recognize other major CN antigens.

Notably, transfer of IgG-depleted, IgM-containing serum in Rag1-deficient hosts resulted in an increase in alveolar macrophage phagocytic index compared to Rag1-deficient mice that received serum lacking both IgG and IgM. No information was provided within this work on whether the IgM-containing serum transfer restored brain control of CN; this will be an important question to be answered in future studies. From these data, the authors suggested a potential role of transferred B-1 cells via secretion of IgM in increasing macrophage uptake of CN as a potential mechanism mediating B cell-dependent protection from CN invasion. Whether uptake by alveolar macrophages represents a conclusive readout for protecting against CN invasion is unclear. Recruited M1 polarized monocyte-derived macrophages are thought to be microbicidal effector cells (often termed inflammatory macrophages “iMac” or exudate macrophages “exMac”), whereas alveolar macrophages have very limited microbicidal activity.^14^ This may be particularly important as non-fungicidal macrophages have been implicated in promoting, instead of restricting, CN dissemination outside the lungs.^15–16^

Therefore, future studies will be required to examine additional measures of lung immunity and to study brain-specific correlates of protection in Rag1-deficient mice following transfer of IgM-containing serum or B-cells in order to further discern the B cell-intrinsic versus antibody-dependent mechanisms of protection. In fact, the results of enhanced pro-inflammatory cytokine production and preserved granulomatous architecture in the CN-infected lungs of some Rag1-deficient mice after B-cell adoptive transfer indicates that a broader investigation of cytokine/chemokine production and of immune cell recruitment may shed further light into the role of B-cells beyond antibody secretion in accounting for the observed protection against CN invasion. Importantly, the inability of Rag1-deficient mice to control fungal proliferation locally in the brain between weeks 2 and 5 post-infection points (Figure 1B) toward a role of lymphocytes (including B-cells) in priming local brain immune responses via microglial activation, not just in preventing dissemination from the lungs into the brain. Hence, future studies will be useful to determine the impact of B-cell or IgM-containing serum transfers on local brain anti-CN host responses by microglial cells. Lastly, it will be of interest to determine whether B-cells protect against lung and brain invasion following infection with other CN strains of different molecular types and with *Cryptococcus gattii*, which has a propensity for pulmonary, not brain, disease in mice and humans.^17^

In summary, the study by Dufaud and colleagues provides the most direct evidence thus far regarding the role of B-cells in controlling CN invasion of the brain. This important study paves the path for additional mechanistic studies that will shed further light on the tissue-specific and antibody-dependent and –independent roles of B-cells in antifungal immunity. These studies may collectively help to design B cell-dependent therapeutic strategies to combat this devastating infection, which based on the results of the present study may be amenable and effective even in patients who lack CD4+ T-cells, that is, those at the highest risk of developing cryptococcosis.
